# Demographic profile and ocular characteristics of stage 5 retinopathy of prematurity at a referral center in Northwest China: implications for implementation

**DOI:** 10.1186/s12886-018-0975-z

**Published:** 2018-11-29

**Authors:** Guo-rui Dou, Man-hong Li, Zi-feng Zhang, Yi-na Lu, Yan-ni Zhu, Hai-yan Wang, Jing Wang, Xiao-jie Wang, Jing Fan, Yu-sheng Wang

**Affiliations:** Department of Ophthalmology, Eye Institute of Chinese PLA, Xijing Hospital, Fourth Military Medical University, Changle West Road 127#, Xi’an, 710032 China

**Keywords:** Retinopathy of prematurity, Stage 5, Retinal detachment, Implementation

## Abstract

**Background:**

Severe retinopathy of prematurity (ROP) with extremely unfavorable prognosis among infants can do great damage to individuals and bring tremendous social-economic burden. The purpose of this study is to describe the demographic and ocular characteristics of infants who presented with stage 5 ROP in order to identify reasons why they have become blind, and to identify contributing factors in order to focus great attention on the current ROP program and to inspire more effort in ROP screening in middle income countries.

**Methods:**

A retrospective review of consecutive infants with stage 5 ROP from December 2010 to December 2016 in Department of Ophthalmology, Xijing Hospital. Various parameters retrieved included birthweight, gestational age, age at initial examination, postmenstrual age, screening details, check-up details and reasons for consultation. Ocular findings were recorded and also detected by ultrasonography.

**Results:**

A retrospective review of 20 consecutive infants with stage 5 ROP are included. Mean birthweight was1712.3 ± 512.97 g and mean gestational age at birth was 32.1 ± 2.21 weeks. Median age at first consultancy was 9.7 month. Median postmenstrual age first consultancy was 52 weeks. All infants were never screened for ROP before they came to the referral center. Of twenty stage 5 ROP infants, 13 cases presented with bilateral stage 5 features. Of the 40 eyes of 20 infants, 33 eyes were diagnosed as stage 5. Leukocoric pupil, closed funnel configuration of retinal detachment (RD), posterior synechia, extraretinal fibrovascular proliferation and retinal folds were the most significant indicators of bad prognosis. Ten eyes appeared no fixation to light, while 30 eyes exhibited following to light or following to toys.

**Conclusions:**

Our study shows that in relatively less-developed regions of China, more needs to be done to spread awareness about the disease among pediatricians, neonatologists and ophthalmologists as well as parents of premature infants. Thus, a comprehensive control system which is a whole network of propaganda, screening, treatment and follow-up are encouraged especially in less developed areas in China as well as worldwide.

## Background

Retinopathy of prematurity (ROP) is a potentially blinding ocular disease affecting premature infants of low birthweight and young gestational age. It is of noted that, despite improvements in early detection and treatment, ROP remains to be a leading cause of childhood blindness and can lead to lifelong visual impairment among infants in both middle income countries and industrialized countries [1–4]. Actually, severe ROP with unfavorable stages leading to serious visual loss could be avoidable in many developed countries, due to strict adherence to ROP screening guidelines, prompt treatment if necessary, and a specialized neonatal intensive care unit with experience of very immature babies [1, 3, 5]. In addition, awareness among pediatricians, neonatologist and parents of premature infants is of great significance in early detection and consultancy.

The “first epidemic” of ROP occurred during the late 1940s and 1950s in Europe and North America as the survival rate of premature babies increased. The major reason is the use of unmonitored supplemental oxygen. Since then, increased awareness of the importance of monitoring blood gases has resulted in a lower incidence of potentially blinding ROP. As neonatal care improved over the next few decades, smaller and less mature babies survived and ROP blindness began to re-emerge (the “second epidemic of ROP”). In last decade, a “third epidemic” of ROP, is said to be occurring in middle-income countries and urban areas of low-income countries like China and India where economies improve neonatal survival with the associated risk of ROP occurrence [2, 6]. Recent estimates suggest that 20,000 (15,500–27,200) survivors of preterm birth become blind from ROP each year with the largest number being in Asia [7, 8]. It indeed has aroused great attention by these countries including Chinese government. The current ROP screening guidelines recommended by the Ministry of Health in 2004 in China were further improved in 2014 [9], which specify that infants who meet the following criteria need to be screened for ROP: Birthweight (BW) of less than 2000 g or gestational age (GA) of less than or equal to 32 weeks [10]. With more effort taken into this screening guideline, the incidence of ROP declined from 2000 to 2012 in developed regions of China such as Beijing, Shanghai, and Guangdong Province [11]. The proportion of infants with Stage 4 and Stage 5 ROP and ROP blind decreased statistically significantly over time after screening guidelines for ROP were issued in 2004 [12].

However unfortunately, the rate and incidence of ROP is still relatively high in districts like Qinghai Province and Gansu Province especially in northwest areas [13]. We still witness quite a few pediatricians in those areas refer high-risk infants to the ophthalmologist until they or the parents notice leucokoria or other ocular abnormalities, when it is too late to do anything as established complications even blindness has occurred. In present study, we describe the demographic and ocular characteristics of these stage 5 ROP infants to identify underling reasons of late consultancy, and to identify contributing factors in order to focus great attention on ROP screening program and to inspire more effort in ROP screening in middle income countries.

## Methods

### Declaration ethical considerations

The study abided by the principles of the Declaration of Helsinki. The protocol was approved by Xijing hospitals’ Ethics Committees. Written, informed-consent of the parents was obtained prior to taking records on correlation data.

### General data collection

In this retrospective study, we reviewed the hospital records of infants with 5 stage ROP detected by ROP screening in the Dept. Ophthalmology of hospital referred above from January 2010 to Dec 2016. Clinical data included gender, singleton or multiple gestation, GA, BW, birth places, oxygen therapy, blood transfusion, phototherapy, respiratory distress syndrome (RDS), mechanical ventilation, intra-ventricular hemorrhage, sepsis, age and postmenstrual age (PMA) at initial examination, reasons for consultation (referral/brought by parents), parents occupations, screening and treatment details. The information entered in the clinic records was based on the screening findings, neonatal discharge summaries and parents recall.

All data were recorded on a standard form, and entered into a database created in Microsoft Office Excel 2010 (Microsoft, Redmond, WA). Data were analyzed using SPSS version 21 (IBM Corp, Armonk, NY) for windows.

### Fundus examination

All infants underwent both indirect ophthalmoscopy using +20D or + 28D lens and Retcam II [14] (Clarity Medical Systems, Inc., Pleasanton, CA) screening after full dilation of the pupils with tropicamide 0.5%. Prior examination results were routinely reviewed, which enabled the identification of change as well as detecting possible error with the former or current examination results. All infants were screened by 2 trained ophthalmologic attendings with eight-year ROP screening experience; assumed progressive disease or uncertain findings were always double-checked by retina specialists within 2 days. ROP was staged according to the international classification of ROP [14].

### Ultrasonography

All infants underwent A/B scan ultrasonography examination standardly performed by single professional technician and confirmed by two experienced ophthalmologists. The ultrasound probe was gently applied on the closed eyelids, and it was oriented in 12 clock-hour positions with the transducer marker pointing towards the center of the eye. At last three ultrasonography images were taken, and the best images were recorded digitally.

## Results

### Demographics

**Fig. 1:**
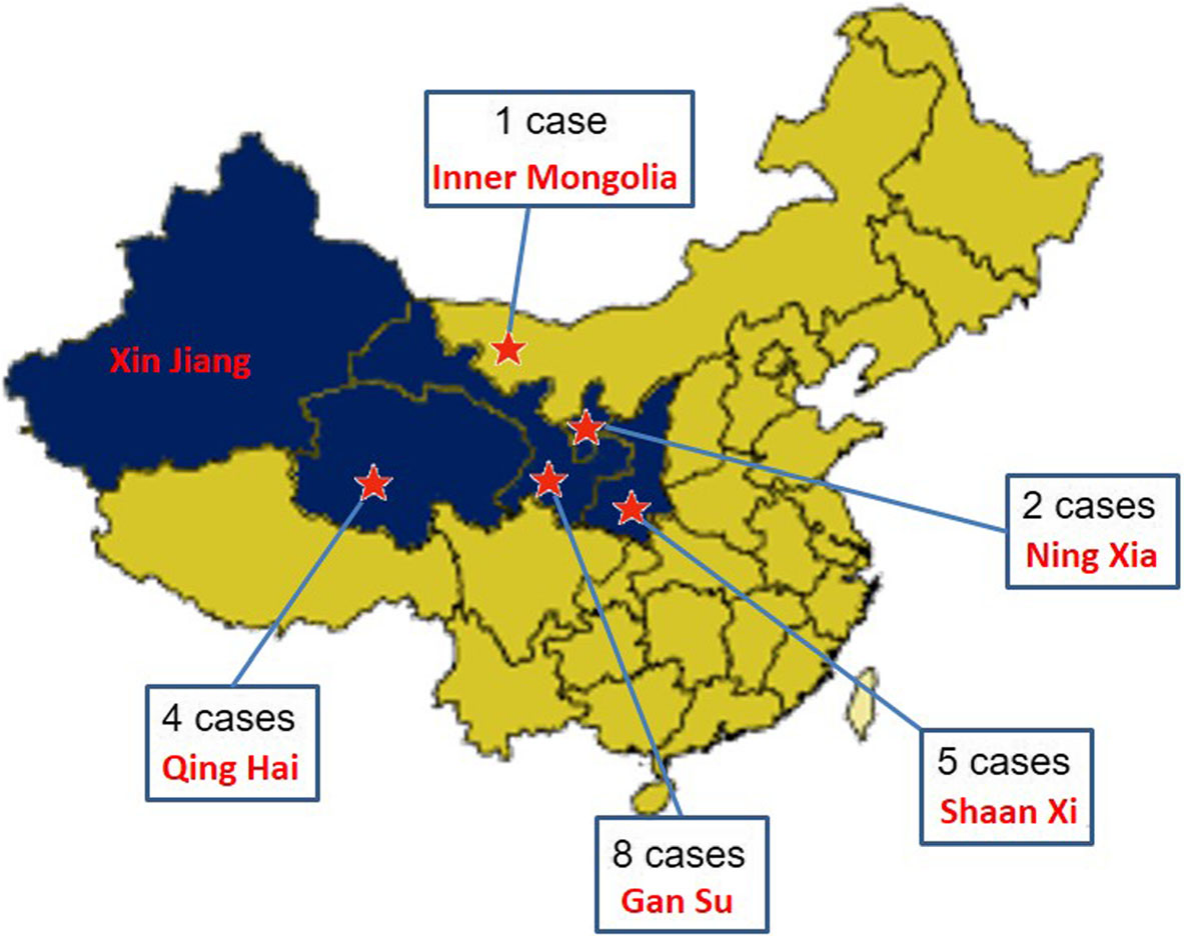
Map showing where 20 children who presented to Xijing Hospital with stage 5 retinopathy of prematurity (ROP) were born. There are five provinces in Northwest China: Shaanxi, Gansu, Ningxia, Qinghai and Xinjiang provinces (blue). The provinces where these stage 5 ROP children were born are indicated by red star. This figure is modified from an original map downloaded from Wikimedia Commons (https://commons.wikimedia.org/wiki/File:China_provinces_shaanxi.png). For this map, the permission is granted to copy, distribute and/or modify this document under the terms of the GNU Free Documentation License, Version 1.2

Twenty infants presented with stage 5 ROP during the study period (Table 1). All infants were outborn (born outside the study center, and 75.0% infants in four neighboring provinces including Gansu, Qinghai, Ningxia and Inner Mongolia, and 25.0% at less developed areas of Shaanxi Province) (Fig. 1). It was of note that infants from Gansu accounted the main parts (8 cases, 40.0%) in the patients. The majority of the 20 infants were born in medical centers of medium size cities or counties regarded as local secondary level facilities which are not equipped with neonatal intensive care units (NICU) facilities or ROP screening (70.0%), and the remainder being born in tertiary hospital. The infants who were received in NICU did not have fundus check-up as these NICU did not have a ROP program. None of these infants was referred by Pediatricians or neonatologists to undertake ROP screening after discharge. Moreover, six (30.0%) cases had been hospitalized for more than 53 days but no ophthalmological consultant was offered meanwhile. The information regarding various parameters was based on neonatal discharge summaries for 12 (60.0%) infants and parents recall for 8 (40.0%) infants. Mean birthweight was 1712 ± 512.97 g (range 890–3000 g) and sixteen (80.0%) infants were < 2000 g (screening criteria currently used in NICU at our institute). Mean gestational age at birth was 32.1 ± 2.21 weeks (range28–36 weeks) and 16 (80%) infants with gestational age in the range of 30–34 weeks. Twelve (60%) infants are single birth. Median age at initial retinal examination was 9.7 month (range, 1.9–53 months) and median post menstrual age (PMA) was 52 week (range, 38–248 weeks) in these 20 infants. There were more male infants (65.0%) (Table 1). All (100%) infants were never screened for ROP (as per recall by parents, neonatal discharge summaries, and ophthalmologist referral notes). Fourteen (70.0%) infants were self-referred (i.e., brought by parents on their own) and ophthalmologists from the infants’ birthplace referred 6 (30.0%), when inability of focusing or abnormal appearance were noticed. Of these14 infants brought by parents on their own, the various reasons for consultation were white reflex in 16eyes (57.1%), involuntary eye movements in 2eye (7.14%) and miscellaneous causes (keratoleukoma) in 4eyes (14.3%).

**Table 1:** Demographic and clinical characteristics

Characteristics	N (%)
Gender	
Male	13 (65.0)
Female	7 (35.0)
Gestational age	
25–29 weeks	2 (10.0)
30–34 weeks	16 (80.0)
> 34 weeks	2 (10.0)
Birth weight (gram)	
< 1000	2 (10.0)
1000–2000	14 (70.0)
> 2000	4 (20.0)
Pregnancy	
Single birth	12 (60.0)
Twin birth	8 (40.0)
Clinical Stages of ROP^a^	
Stage 3	1 (2.5)
Stage 4b	5 (12.5)
Stage 5	33 (82.5)
Regressed	1 (2.5)
Visual function^a^	
No fixation to light	10 (25.0)
Following to light	24 (60.0)
Following to toys	6 (15.0)

^a^calculated based on 40 eyes

### Clinical signs

Among these 20 subjects (40 eyes), ROP was 100% bilateral (for each infant at least one eye had Stage 5 ROP). Bilateral Stage 5 ROP was noted in 13 (65.0%) infants, and only one eye was evaluated with the chance for surgical treatment, while other Stage 5 eyes with no apparent benefit from surgery were considered as advanced untreatable disease (Table 1). Ten eyes appeared no fixation to light, while 30 eyes exhibited following to light or following to toys (Table 1).

**Fig. 2:**
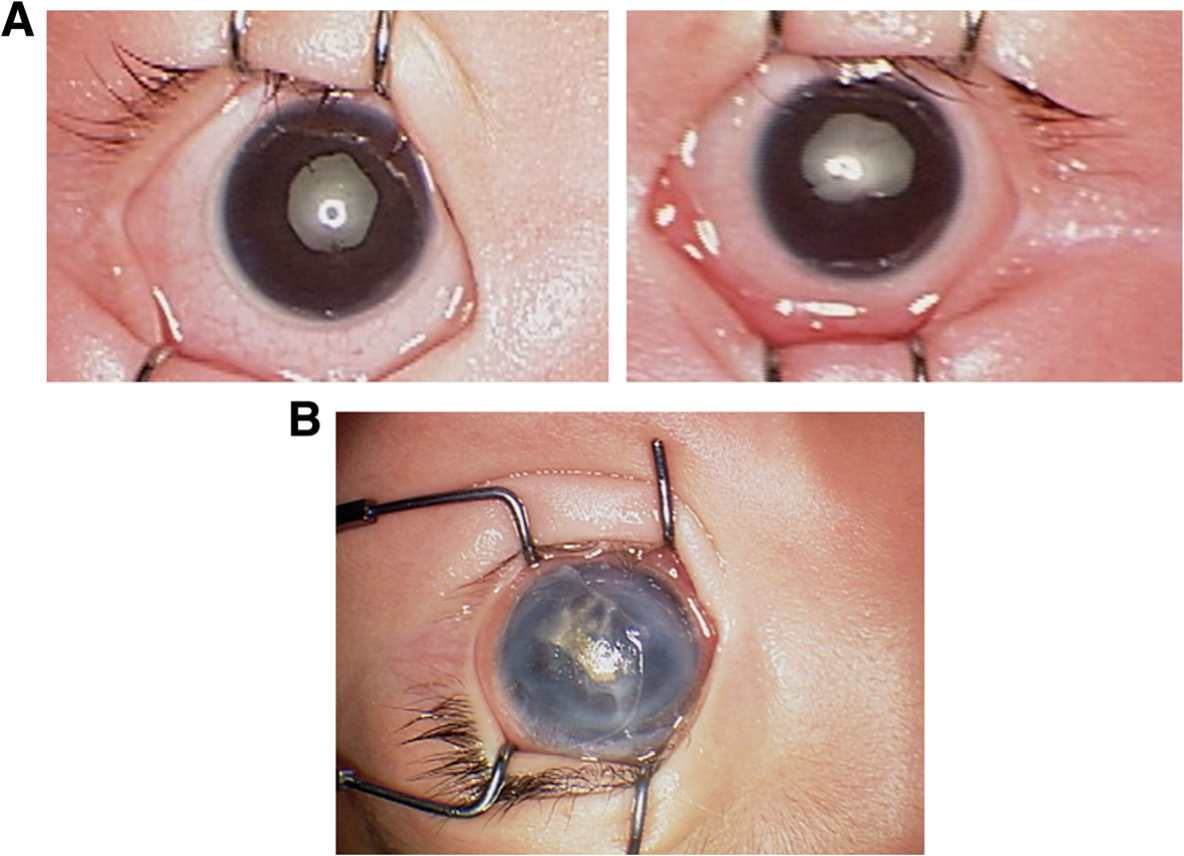
Representative images of retinopathy of prematurity (ROP) complication in anterior segment of the eye. **a**. Leukocoria and posterior synechia in both eyes of one infant, **b**. keratoleukoma in left eye of one case

**Fig. 3:**
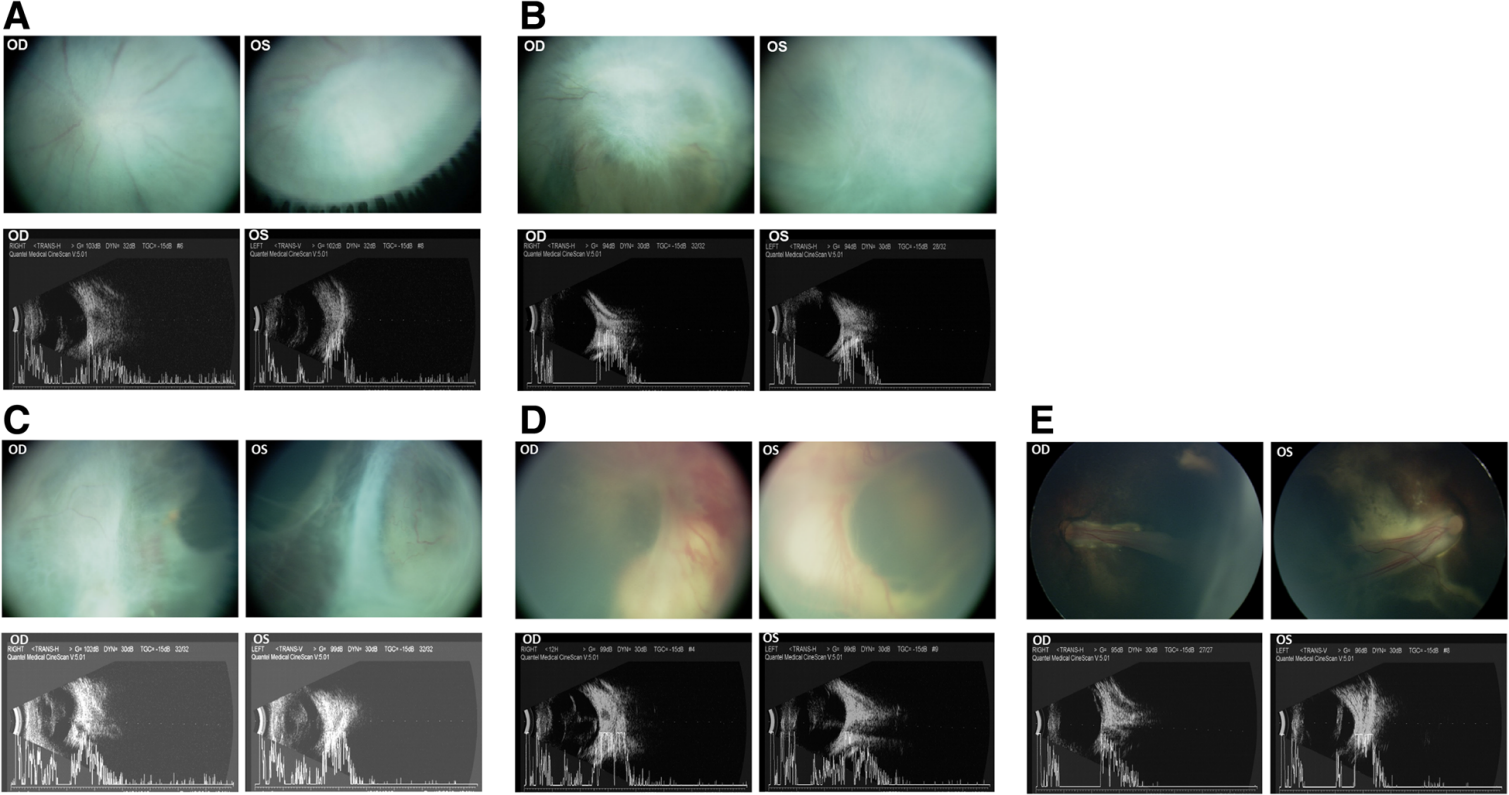
Representative images of retinal detachment (RD) of advanced stages of retinopathy of prematurity (ROP). **a**. A four-month-old boy infant from Inner Mongolia with 1600 g of birthweight. Top image, RD accompanied by peripheral fibrous traction to cilliary body in two eyes of one infant. Bottom image, the ultrasound of the corresponding RD with fibrosis membrane seen extending from peripheral retina to the edge of cilliary body, **b**. A ten-month-old boy infant from Gansu Province with 2100 g of birthweight. Top image, RD accompanied by a thick fibrous traction membrane in two eyes of one infant. Bottom image, the ultrasound of the corresponding RD with fibrosis membrane seen spreading through the whole retina, **c**. A three-month-old boy infant from Gansu Province with 1250 g of birthweight. Top image, RD accompanied by centripetal traction of neuro-sensory retina towards to optic discs along the major vascular arcade and formation of retinal fold. Bottom image, the ultrasound of the corresponding RD, **d**. A two-month-old girl infant from Gansu Province with 2000 g of birthweight. Top image, RD accompanied by fibrous membrane and retinal bleeding. Bottom image, the ultrasound of the corresponding RD, **e**. A six-month-old boy infant from Gansu Province with 1500 g of birthweight. Top image, retina rolled-up as a stalk anteriorly to just behind the lens. Bottom image, the ultrasound of the corresponding RD

Fourteen children (twenty-seven eyes) came with leukocoric pupils (Fig. 2a). Most of the corneas maintain transparency, whereas two cases (two eyes) presented with corneal opacity (Fig. 2b). Posterior synechia was noted in 16 of these 40 eyes (40.0%) (Fig. 2a). Due to the opacity caused by severe leukocoria or keratoleukoma, fundus of 7 eyes could not be absolutely detected by indirect ophthalmoscopy and Retcam II, and were diagnosed as stage 5 by ultrasound scan. In those detectable fundus, closed funnel configuration of retinal detachment (RD) accounted for the main proportion (26 eyes, 65.0%), which were also confirmed by the image from ultrasound scan (Fig. 3) (Table 2). These significant signs of bad prognosis were predominantly presented children at the consultancy age between 16 and 48 weeks (at PMA between 51 and 84 weeks) (Table 3).

**Table 2:** characteristics of ocular signs

Characteristics	Infants/eyes	Percentage (%) ^a^
Leukokoric pupil	14/27	67.5
Corneal opacity	2/2	5.0
Posterior synechia	9/16	40.0
Absolutely undetectable fundus by indirect ophthalmoscopy or Retcam	5/7	17.5
Closed funnel configuration of RD in detectable fundus by indirect ophthalmoscopy or Retcam	16/22	55.0
Closed funnel configuration of RD with retinal fold in detectable fundus by indirect ophthalmoscopy or Retcam	2/4	10.0
RD accompanied with retinal hemorrhage	3/4	5.0
Retinal exudation	2/3	7.5

^a^calculated based on 40 eyes

**Table 3:** clinical characteristics and Age /post menstrual age (PMA) at consultancy

Category(eyes)	Age/PMA at Consultancy (weeks)			
Characteristics	4–8/38–40	8–16/41–46	16–48/51–84	> 48/80–228
Leukokoric pupil	3	4	18	2
Posterior synechia			12	2
Closed funnel configuration of RD	3	4	20	6

In twenty-six eyes with confirmed closed funnel detectable RD, the presentation of fundus showcases the complexity and diversity of severe conditions as seen through both funduscopic examination and ultrasonic finding. For instance, extraretinal fibrovascular proliferation (EFP) fibrous and fibrous membrane varied in the origin, scope, extension and traction. RD with periphery fibrosis was noted in six eyes by both funduscopic examination and ultrasonic finding (Fig. 3a).The fibrous elements originated from periphery and central retina and dragged to ciliary body. In five eyes, total RD was found and a spreading fibrous traction membrane with retinal vascular avulsion was observed (Fig. 3b). Three eyes showed total RD with centripetal traction of neuro-sensory retina along the major vascular arcade. They progressed to a fibrous traction membrane and severe retinal folds were observed along the superotemporal retinal vascular arcade (Fig. 3c). In addition, a dense retinal neovascularization and hemorrhage accompanied RD in 4 eyes with fibrous traction membrane and RD (Fig. 3d). Particularly worth mentioning is in 8eyes, we observed the detached retina rolled-up as a stalk anteriorly to just behind the lens with exposed choroidal capillaries (Fig. 3e). Combined with history of preterm birth, low birthweight, oxygen inhalation, the ocular checkup on their parents, we finally excluded the suspected diagnosis of familial exudative vitreoretinopathy.

## Discussion

ROP is a preventable and treatable cause of childhood blindness if timely screening and treatment are carried out. The screening criteria and treatment guidelines have already been strictly carried out in developed country, thus recent studies reveal that the incidence of ROP in these countries is decreasing [11, 15, 16]. China is currently experiencing the third epidemic of ROP and has gradually been forcing the ROP screening and treatment guidelines. ROP screening program by the Ministry of Health of China has been carried out since 2004. From then, an increasing number of ROP studies were conducted. It has been shown that low number of advanced stage ROP presenting from central and east part of China,probably reflects the fact in eastern China, where the majority of level three neonatal units are located, has a very good ROP program [6, 12]. However, the incidence of ROP varied between regions, and certain severe cases still can be witnessed in less developed areas of China [17]. This retrospective analysis demonstrated a series of stage 5 ROP infants with extremely unfavorable prognosis. Twenty infants with end stage ROP presented in the largest tertiary hospital in northwestern China in over a total recruitment period of 6 years i.e. an average of 3–4 infants per year.

Owing to the uneven development of regional economies in China, the central and west regions, particularly the latter, now, in general, lag behind the coastal areas in east China. In other words, the prenatal care, delivery care, and postnatal care, as well as general socioeconomic and educational conditions, have distinct disparities among regions. The incidence of ROP in different regions of China varies from 10.03–23.3% according to previous reports [10]. It is notable that, except Shaanxi Province, in other relatively undeveloped provinces in northwestern China, the ROP screening centers has not been widely available due to the relatively economic, cultural backwardness and less social initiatives. The observation that major proportion of cases are from Gansu Province might due to its proximity to Shaanxi. It is highly possible that the survival rate of premature infants is low or parents from remote areas like Xin Jiang Province may give up healthcare. We show that children in this group came to first ophthalmologic consultancy when they were several months old, which implies, for these eligible infants, the missing of the best time-window of laser or surgical intervention.

Several epidemiological ROP studies have reported stage 4 or 5 incidences of 0.6–12.3% in preterm infants born at a GA of 26 weeks or before [18]. These highly advanced stages seem to occur very rarely in most developed countries, and even could not be observed according to a large high-risk German cohort [5]. However, in accordance with our report, groups from North India and Mexico, the counties also involved in 3rd epidemic of ROP, reported similar epidemiological stage 4b and stage 5 ROP preterm infants [19, 20]. A common and important finding of this current study and the Indian group was none of these infants’ parents was referred or offered for retinal examination of their premature babies either in the NICU or after discharge from hospitals. It has been demonstrated that significant difference between infants who had been recommended to undergo ocular examination in early birth age and those who had not, indicating the vital role of pediatricians and neonatologists in the timely informing and referring premature infants to professional consultancy in order to decrease its burden of complications [21]. Therefore, although Early Treatment For Retinopathy of Prematurity Cooperative (ETROP) [18] has given clear criteria for early management of high-risk eyes of premature, degree of awareness regarding ROP among pediatricians and neonatologists closely relates to the early diagnosis and prevention from delayed examination. Clear legislation and protocols are required for relative personnel including pediatricians and neonatologist, who identify infants requiring examination, to further inform the necessity of screening to the parents and refer them to ophthalmologists.

Awareness from parents of premature infants is also of great significance in early detection and consultancy. In our series cases, most infants presented at 16–48 weeks only until parents noticed the abnormal appearance like leucokoria or cornea opacity. Thus, likely reasons for the late consultancy include no knowledge of the ROP risk and overlook on the vision problems from infants’ parents. Therefore, medical staff should keep in mind that it is their responsibility to sufficiently inform the new parents of the basic neonatal visual knowledge as well as the ROP risk. A vision screening guideline from American Association for Pediatric Ophthalmology and Strabismus or recommendation statement of Vision Screening in Children Aged 6 Months to 5 Years is recommended to parents, pediatricians, neonatologists [22]. We also noticed in our data that advanced ROP boys outnumbered girls, which may reflect gender differences in the incidence and severity of ROP. As the sex discrimination still exists in poor rural areas of China and boy probably could be paid more attention, we assume there may be difference in health seeking behavior. Moreover, once the neonate is regarded as a ROP suspect, the compliance on a regular and diligent follow-up examination is believed to account an essential part in fighting with ROP associated blindness [23, 24]. It is a joint effort for the medical personnel and parents to ascertain the status of premature infants and impel them on a regular follow-up until risk obliteration.

In this current study, four ROP infants were with BW (> 2000 g). Only one of these four experienced ARDS, while parents of the other three recalled the natural birth. The possibility that the unmonitored supplemental oxygen may be applied during the hospital stay could not be excluded. According to an Indian study, most of more bigger and mature babies had received unmonitored oxygen therapy [25]. In NICU unit, oxygen therapy should be appropriate and restricted adopted to reduce the incidence and severity of ROP without unduly increasing death rates. Besides, higher BW infants who develop ROP may have some genetic alteration, which requires further investigations to determine the role of genetics. From the previous reports, we could see the varied incidence of ROP between countries and even between regions. Accordingly, the ROP screening criteria differ from one population to another [10]. Slightly different with US and UK criteria, the guidelines used in China include more mature infants, in order not to miss infants who may need treatment. Even so, in China, several reports on ROP screening included neonates with GA ranged from 32 weeks to 37 weeks or BW between 1500 g and 2000 g, and specified that more mature babies can develop severe ROP [6, 7, 13, 26]. Chen et al. [27] suggested in China infants with BW ≥ 2000 g should be examined at three weeks after birth. Thus, considering the fact that the range of GA and BW in ROP patients in developing countries is wider than those in developed countries, appropriate expanding on the screening criteria should be considered on the big infants with potential risk factors.

For advanced stage ROP, the utility of the combining funduscopy or Retcam II with ultrasonography could display more details. Especially for stage 5 ROP which cannot be detected by funduscopy, ultrasonography offers a more detail and visualized configuration information [28]. For the 33 eyes with stage 5 ROP in our report, only 26 could be visualized by funduscopy or Retcam II. Moreover, the use of ultrasonography is advantageous for premature neonates with poor pupillary dilation and opacity of refractive media. It has been accepted that scleral buckling and vitrectomy could be used to manage advanced ROP [1, 18, 29]. However, most studies revealed that anatomical and visual outcome by these surgical interventions is very poor for stage 5 ROP [30, 31]. A few cases showed posterior pole reattached but had complete re-detachment in the later follow-up. The retina of eyes at stage 5 ROP is vulnerable to a recurrence of the RD after being attached by vitrectomy [32].Surgical outcome was particularly poorest in narrow–narrow configuration, the extremely unfavorable situation similarly observed in our cases. The consequently poor outcome reemphasize the need for increasing ROP awareness among the medical facilities in order to reduce the shown up of advanced ROP.

Our hospital is a tertiary referral center in Northwest China that receives patients mainly from adjacent relatively undeveloped regions, and those presenting here may not be representative of all babies with severe ROP in Northwest area, which is a major inherent shortfall of this study. Here in this present study, we showed a series of unfavorable stage 5 ROP without timely consultancy. We also encountered 13 cases with bilateral stage 5 ROP which is equivalent to blindness. We assume more similar cases may exist in actual life. Some children with severe ROP may have been referred directly to hospitals in Beijing and Shanghai, or some in the highly poor areas may give up seeking for medical treatment, which would bias the data in a limited number of cases. However, this current study in some ways do reflect alarming reality of ROP program in China as well as in other countries with unbalanced regional development, and urgent requirement on collaboration between pediatricians, neonatologists, ophthalmologists, and allied health personnel, together with parents.

Several studies have emphasized that a remarkable portion of ROP-related blindness is preventable in the presence of a structured screening program and by increasing awareness of ROP in physicians and parents [15, 33]. Combining the conditions of unbalanced regional development, ROP programs should have different content and emphasis according to whether the setting is in an economically advanced or developing area [34]. Telemedicine could be an underlining promising approach. Trained neonatal personnel could capture images and clinical data from infants, which would subsequently be interpreted by a remote ROP expert. This might improve the quality, accessibility and cost of ROP care [35]. However, input on facilities and experienced staffs are basic requirements. A “three grades network of prevention and treatment of ROP” might potentially be more realistic and cater to the increasing needing for ROP screening and professional care in relatively undeveloped areas [36]. Primary medical centers at the county level form the primary unit to be responsible for proper neonatal care and referring ROP suspects to ROP screening; Ophthalmology department in general hospital or maternal and child care service centers in prefecture-level cities constitute the secondary unit to mainly carry on the ROP screening, follow-up as well as referring infant in need of treatment to upper level; Major medical centers in large cities which are capable to treat severe ROP constitute the tertiary unit; therefore, this tentative plan not only caters to the social development, but also avoids the waste of resource.

## Conclusions

As a comprehensive center for ROP screening and treatment in northwestern China, we still witness a series advanced stage ROP infants with extremely bad prognosis. The incidence of ROP varied between regions and certain frustrated cases still can be witnessed in less developed areas of China. Thus, in order to prevent blindness due to ROP, we must improve the implementation of ROP program in unbalance developed regions.

## Abbreviations

BW: Birthweight
EFP: Extraretinal fibrovascular proliferation
ETROP: Early Treatment for Retinopathy of Prematurity
GA: Gestational age
NICU: Neonatal intensive care units
PMA: Post menstrual age
RD: Retinal detachment
RDS: Respiratory distress syndrome
ROP: Retinopathy of prematurity
